# Calcitonin gene-related peptide is a key factor in the homing of transplanted human MSCs to sites of spinal cord injury

**DOI:** 10.1038/srep27724

**Published:** 2016-06-14

**Authors:** Yu Zhang, Jinhua Yang, Peng Zhang, Tao Liu, Jianwei Xu, Zhihai Fan, Yixin Shen, Wenjie Li, Huanxiang Zhang

**Affiliations:** 1 Department of Orthopedics, the Second Affiliated Hospital of Soochow University, Suzhou, China; 2 Department of Orthopedics, the Second People’s Hospital of Changshu, Suzhou, China; 3 Department of Cell Biology, Jiangsu Key Laboratory of Stem Cell Research, Medical College of Soochow University, Suzhou Industrial Park, Suzhou, China; 4 Department of Human Anatomy, Medical College of Soochow University, Suzhou Industrial Park, Suzhou, China

## Abstract

Mesenchymal stem cells (MSCs) can be used to treat many diseases, including spinal cord injury (SCI). Treatment relies mostly on the precise navigation of cells to the injury site for rebuilding the damaged spinal cord. However, the key factors guiding MSCs to the epicenter of SCI remain unknown. Here, we demonstrated that calcitonin gene-related peptide (CGRP), a neural peptide synthesized in spinal cord, can dramatically aid the homing of human umbilical cord mesenchymal stem cells (HUMSCs) in spinal cord-transected SCI rats. First, HUMSCs exhibited chemotactic responses *in vitro* to CGRP. By time-lapse video analysis, increased chemotactic index (CMI), forward migration index (FMI) and speed contributed to this observed migration. Then, through enzyme immunoassay, higher CGRP concentrations at the lesion site were observed after injury. The release of CGRP directed HUMSCs to the injury site, which was suppressed by CGRP 8–37, a CGRP antagonist. We also verified that the PI3K/Akt and p38MAPK signaling pathways played a critical role in the CGRP-induced chemotactic migration of HUMSCs. Collectively, our data reveal that CGRP is a key chemokine that helps HUMSCs migrate to the lesion site and thereby can be used as a model molecule to study MSCs homing after SCI.

Spinal cord injury (SCI) is a devastating disease that has significant human and societal costs^1^. Stem cell transplantation provides hope for therapeutic repair after SCI^2^. With the advantages of fewer ethical concerns and less immune rejection^3^,^4^,^5^,^6^, mesenchymal stem cells (MSCs) appear to exert neuroprotective effects through the paracrine action of various cellular factors and compensate for neurological deficits after their differentiation to neurocytes^7^,^8^,^9^,^10^. Among the MSCs, human umbilical cord mesenchymal stem cells (HUMSCs) can be easily obtained from noninvasive collection methods and cultured with minimal manipulation. Additionally, they are rich in stemness with a broad and efficient differentiation potential^11^.

Currently, stem cell transplantation for SCI is commonly implemented by direct delivery into the injured parenchyma^12^. However, many disadvantages accompany this method, including additional trauma in intramedullary transplantation surgery and the loss of grafted cells in a hostile environment, but intrathecal transplantation by lumbar puncture (LP) and intravascular delivery are minimally invasive techniques. Considering the impenetrable blood-brain barrier, LP provides ideal access for cellular transplants with higher efficiency and less host immune response^13^,^14^. Regardless of the method of administration, MSCs home to the lesion site and exert protective effects. The supposed mechanism of homing involves the release of multiple chemokines at the injury site to recruit the transplanted stem cells^15^.

Calcitonin gene-related peptide (CGRP) is a 37-amino acid peptide mainly synthesized in dorsal root ganglion (DRG) and in the ventral horn of the spinal cord^16^,^17^. It is transported along axons to the peripheral and central endings of sensory nerve fibers throughout the body, with the highest concentrations in the spinal cord^18^,^19^,^20^,^21^. CGRP plays a well-recognized and pivotal role in repairing tissue, activating its cognate G protein-coupled receptor to initiate intracellular signaling^22^,^23^. Following tissue injury, such as SCI, increased CGRP participates in vasodilation, sensory transmission, immune modulation and nerve regeneration^24^,^25^,^26^,^27^,^28^. Recently, MSCs were found to express CGRP receptors on the cell membrane surface^29^,^30^. Although numerous studies have characterized the stimulatory differentiation effects of CGRP on MSCs^31^, no studies have explored CGRP-induced chemotactic migration in HUMSCs.

In the present study, HUMSC chemotaxis in the presence of CGRP concentration gradients was first confirmed *in vitro*. More HUMSCs migrated trans-filter in response to CGRP than control cells without CGRP. Detailed time-lapse video analysis indicated the potential involvement of increased speed, chemotactic index (CMI) and forward migration index (FMI) in this chemotactic migration. Annexin-V/PI and CCK-8 assays clarified that CGRP had no effects on HUMSCs proliferation, apoptosis and death. Based on these results, we further speculated that the homing of HUMSCs to the lesion site was promoted by secreted CGRP after SCI. Experiments in both the contusion and transection groups caused CGRP enhancement at the surgery site (T9). With the disruption of the meninges, the released CGRP attracted large numbers of HUMSCs to the injury site, which could be reduced by the specific CGRP antagonist CGRP 8–37. We also demonstrated that PI3K/Akt and p38MAPK signaling were involved in this process as blocking these signaling pathways decreased the number of cells that migrated in response to CGRP.

## Materials and Methods

### Isolation and culture of HUMSCs

Experiments involving human subjects were carried out in accordance with the Code of Ethics of the World Medical Association (Declaration of Helsinki). The Research Ethics Board of Soochow University approved all experimental protocols. HUMSCs were generously donated, with appropriate ethical approval, from the Stem Cell and Tissue Engineering Research Center, Gui Zhou Medical University, China. With written informed consent from all donors, fresh human umbilical cords were obtained after birth and immersed in D-Hanks’ balanced salt solution (DHBSS) at 4 °C. The umbilical cord vessels and outer membrane were removed while still in DHBSS. Then, mesenchymal tissue in Wharton’s jelly was diced into 1-cm^3^ cubes and inoculated in 75-cm^2^ culture flasks. The flasks were filled with standard medium composed of low-glucose Dulbecco’s modified Eagle’s medium (L-DMEM, Gibco, Gaithersburg, MD) with 10% fetal bovine serum (FBS, Gibco) and 1% penicillin-streptomycin and incubated in a 5% CO_2_ atmosphere at 37 °C. Half of the media was replaced for the first 3 d, and then, complete media replacement occurred every 3 d. When HUMSCs reached 50% confluence, tissue cubes were removed from the culture bottles. HUMSCs at 80–90% confluence were dissociated with a 0.25% trypsin-EDTA solution (Sigma, St. Louis, MO) and subcultured at a ratio of 1:2. HUMSCs at passages 3–10 were used for experiments. The cells expanded *in vitro* were CD90^+^, CD44^+^, CD105^+^, CD73^+^ and NOT-HUMSCs^−^.

### Migration assays

#### Boyden chamber

After a 30-min starvation period in serum-free medium, HUMSC migration was assessed with CGRP concentration gradients using a 48-well modified Boyden chamber (Neuro Probe, Gaithersburg, MD). Briefly, a 50-μL MSC suspension (5 × 10^4^ cells/ml) was seeded in the upper chamber of each well on a poly-L-lysine (PLL; Sigma)-precoated PVP-free polycarbonate membrane (8 μm pore size; Osmonics, Minnetonka, MN). Then, 30 μL L-DMEM containing rat CGRP (Sigma), ranging from 10^−9^ mol/L to 10^−6^ mol/L, was added to the lower compartment. The apparatus was incubated for 6 h at 37 °C in a 5% CO_2_ humidified incubator. After migration, the cells on the upper side of the filter were removed. After fixation in 4% formaldehyde in 0.1 mol/L phosphate-buffered saline (PBS) (pH 7.2), cells attached to the lower side were stained for 30 min in 0.1% cresyl violet and counted at 100× magnification in all fields of each well. To verify the effects of various inhibitors on HUMSC migration, cells were pretreated with 10^−6^ mol/L CGRP 8–37 (Rat, Tocris, USA), 3 × 10^−5^ mol/L LY294002 (Promega, Madison, WI), 3 × 10^−5^ mol/L SB203580 (Alexis Biochemical, Lausen, Switzerland), 5 × 10^−5^ mol/L PD98059 (Enzo, London, UK), or 10^−5^ mol/L SP600125 (Enzo) for 30 min before the migration assay. The same concentration of inhibitors was present in the upper chambers of the Boyden chamber, with 10^−6^ mol/L CGRP in the lower chambers, during the migration assays.

#### Dunn chamber

MSC chemotaxis (after a 30-min starvation period in serum-free medium) was directly viewed and analyzed in stable CGRP gradients using a Dunn chamber (Hawksley, Lancing, UK), which was made from a Helber bacteria counting chamber by grinding a circular well in the central platform to produce a 1-mm-wide annular bridge between the inner and the outer wells. Once added to the outer well of the device, chemotactic factors diffuse across the mid-bridge to the inner well and form a concentration gradient. This apparatus allows for the direct monitoring of cell locomotion and the intuitive analysis of migration speed, turning behavior, and migration directionality. We placed a 24 × 24 mm inverted coverslip on the chamber, ensuring that the surface coated with cells was facing down, and cell motion was recorded on the annular bridge between the concentric inner and outer wells. Here, the outer well of the Dunn chamber was filled with medium containing different CGRP concentrations, and the concentric inner well was filled with only L-DMEM. For blocking experiments, HUMSCs were pretreated with 10^−6^ mol/L CGRP 8–37 for 30 min, and then medium with an inhibitor was added to both Dunn chamber wells; 10^−6^ mol/L CGRP was also added to the outer well. Cells loaded onto the Dunn chamber were captured every 5 min using a 10× objective with a Leica DMI 6000 B microscope for 6 h at 37 °C and 5% CO_2_. The chemotactic index (CMI), which represents the number of cells migrating in the direction of CGRP to the total number of cells, was used to examine the chemotactic sensitivity to CGRP. To determine the efficiency of forward migration during the recording period, the forward migration index (FMI) was calculated as the ratio of forward progress (net distance the cell migrated toward the CGRP source) to the total path length (total distance the cell traveled in the field). Meanwhile, cell speed was obtained from each time lapse interval recorded during the 6-h period.

### Cell proliferation and cytotoxicity assay

HUMSCs (2000 cells/well) were seeded in a 96-well plate, grown in L-DMEM containing 10% FBS and cultured in an incubator containing 5% CO_2_ at 37 °C. The different added substances were as follows: Control group: 0.1 mol/L PBS, CGRP group: 10^−6^ mol/L CGRP, CGRP 8–37 group: 10^−6^ mol/L CGRP 8–37, LY group: 10^−6^ mol/L CGRP + 3 × 10^−5^ mol/L LY294002, SB group: 10^−6^ mol/L CGRP + 3 × 10^−5^ mol/L SB203580. Medium was replaced every 3 d. Removed supernatant on the 7th day. Cells were washed twice with PBS. 100 μL solution composed of 10 μL CCK-8 (Sigma) and 90 μL L-DMEM were added to each well. After an incubation of 2 h, absorbance at 450 nm and a reference at 650 nm was measured using a microplate reader (BieTek). In each experiment, measurements were repeated in three independent experiments.

### Flow cytometry detection of apoptosis and death

Cell apoptosis and death were quantified with double staining of fluorescein isothiocyanate (FITC) conjugated Annexin-V and propidium iodide (PI; Biouniquer, BU-AP0103). HUMSCs were seeded at a density of 2 × 10^4^ cells/cm^2^ in a 6-well plate, and nourished in L-DMEM containing 10% FBS and certain concentration of substances (same as above) for 3 d. Then the medium was totally replaced. After a 4 d maintenance, freshly trypsinized cells combined with cells in supernatant were pooled, washed twice with PBS, and processed following the manufacturer’s instructions. Ten thousand cells per sample were acquired with a FACS flow cytometer (FACScan). Cells fluorescence was analyzed with flow cytometry using the Cell Quest Pro software (Beckman Coulter). Assays were performed in triplicates and repeated in three independent experiments.

### Cell labeling

For accurate quantification of transplanted HUMSCs *in vivo*, cells were labeled with Hoechst 33258 pre-transplantation. Briefly, 10 mg/L H33258 was added to the culture wells for 2 h. Then, medium was discarded, and cells were washed twice with PBS. Afterwards, HUMSCs were cultured normally in standard medium until transplantation.

### Western blot analysis

Western blot procedures were performed as previously described^32^. Briefly, cells that had been induced by different CGRP concentrations for 6 h were lysed with a protein extraction reagent. Lysates were centrifuged at 12,000 rpm at 4 °C for 5 min to remove cell debris. The supernatants were transferred to fresh tubes, and protein concentrations were measured with a BCA assay kit (Applygen). Identical amounts (15–25 μg) of protein lysates were separated by electrophoresis using 10% SDS-PAGE gels and then transferred to 0.45-mm nitrocellulose membranes (Millipore) using a Trans-Blot SD Semi-Dry Electrophoretic Transfer Cell (Bio-Rad). After blocking with 5% nonfat milk in TBST (0.1 mol/L Tris-HCl, pH 7.4, 0.15 mol/L NaCl, with 0.1% Tween-20), the membrane was incubated with the following primary antibodies (rabbit monoclonal; 1:1,000) raised against phospho- or nonphospho-protein kinases overnight at 4 °C: antiphospho-ERK1/2 (Thr202/Tyr204), anti-ERK1/2, antiphospho-Akt (Ser473), anti-Akt, antiphospho-SAPK/JNK (Thr183/Tyr185), anti-SAPK/JNK, antiphospho-p38MAPK (Thr180/Tyr182), and anti-p38MAPK (all from Cell Signaling Technologies). Membranes were then washed 3 times with TBST and incubated for 1 h at room temperature with the appropriate HRP-conjugated secondary antibodies (1:2,000 dilution; Cell Signaling Technologies). Thereafter, the antigen-antibody complexes were visualized by ECL (Biological Industries). The intensity of protein bands was quantified by gel image analysis software (Image Lab 4.1).

### Animals

All animal experiments were conducted in accordance with Chinese laws and as previously approved by the Soochow University Veterinary Authority. Five-week-old adult female Sprague-Dawley rats (180–220 g) were purchased from the Experimental Animal Center of Soochow University of China.

To test CGRP concentrations, 3 rats were randomly selected from 33 rats in the pre-operation group (without any surgery). The other 30 rats were divided into the contusion and transection groups, which were sacrificed at 2 h, 1 d, 3 d, 7 d, 14 d post-surgery (n = 3 for each time point/group). For cell transplantation experiments, 20 rats were randomly allocated into 4 groups (n = 5 per group). Groups (1–3) had a T9 transection followed by HUMSC transplantation through LP at L3-4 but with different methods of administration at the lesion site: (1) the control group, with continuous PBS administration; (2) the CGRP 8–37 group, with the injection of 10^−6^ mol/L CGRP 8–37; (3) the CGRP group, with the administration of 10^−6^ mol/L CGRP to the injury site; and (4) the contusion SCI group, with a T9 clamping contusion but without tearing the dura and the remaining procedures similar to the control group.

### SCI modeling and HUMSC grafting

Rats were anesthetized using an intraperitoneal injection of chloral hydrate. After exposure by laminectomy at the T8-T9 vertebral level, the spinal cord was completely transected using a surgical blade (transection group) or was crushed with a modified aneurysm forceps (FE752K, YASARGIL, Germany) for 1 sec, which exerted 180 g (approximately 1.77 N) closing force (contusion group). A 3-mm^3^ gelatin sponge was placed into the fenestra for adequate hemostasis. Then, an indwelling flexible tube with a 2-mm inner diameter was placed at the injury site, which allowed us to inject different agents continuously every day. The overlying muscle and skin were sutured with 3–0 nylon. Three days after SCI, rats were anesthetized as described above and placed prone, and HUMSCs were transplanted via LP. A small longitudinal incision was made over the L3-4 spinous processes, and the skin was retracted. A 50-μL microsyringe needle was advanced into the spinal canal at L3-4. Approximately 2 × 10^6^ HUMSCs diluted in 40 μL PBS were slowly injected into the intrathecal space, and 10 μL PBS was used to flush the needle. To prevent backflow of the injected contents, the LP needle remained in place for at least 2 min and then was slowly withdrawn; after which, the skin was sutured with 3–0 nylon. During the 7 d post-transplantation, 100 μL PBS, CGRP 8–37 or CGRP was slowly injected daily at the lesion site, accompanied by sealing the tube to prevent backflow and infection.

### Tissue preparation

For the ELISA assay, rats were deeply anesthetized as described above at different times. After perfusion with 0.1 mol/L PBS, spinal segments 5 mm in length were excised, encompassing the lesion site at T9, the cervical region C7 and the caudal site L1. The tissue segments were rapidly frozen with liquid nitrogen and stored at −80 °C. Cold PBS (1:9, pH 7.4) was added, and the samples were homogenized with a grinder (IKA T10, Germany) and centrifuged for 20 min at 2,000–3,000 rpm. The supernatants were collected and stored at −20 °C until testing. For immunohistochemistry, rats were anesthetized 7 d after HUMSC grafting and then perfused with 4% paraformaldehyde and 0.1 mol/L PBS. The same segments as those mentioned above were obtained followed by further fixation with 4% paraformaldehyde and dehydration with 30% saccharose. Samples were cryosectioned sagittally into 20-μm sections to examine the distribution of transplanted cells.

### ELISA

CGRP in the parenchyma of spinal cord segments at various time points (pre-operation and 2 h, 1 d, 3 d, 7 d, and 14 d post-surgery) and different sites (C7, T9, and L1) was detected with a rat CGRP enzyme immunoassay kit (SPIBIO, France) according to the manufacturer’s specifications.

### Immunohistochemistry

Tissue sections for fluorescence immunohistochemistry were rehydrated in 0.1 mol/L PBS, blocked in 3% bovine serum albumin (BSA) for 1 h, and incubated with primary antibodies overnight at 4 °C. The primary antibodies were diluted in PBS with 0.02% NaN_3_, 3% BSA, and 0.2% Triton X-100 at the following working concentrations: rabbit monoclonal anti-HLA, 1:200 dilution (Abcam); mouse monoclonal anti-GFAP-Cy3-Conjugate 1:1,000 dilution (C9205, Sigma). After incubation with primary antibodies, sections were rinsed with PBS prior to secondary antibody application. An Alexa Fluor 488 donkey anti-rabbit IgG (Invitrogen) was diluted 1:500 in PBS with 0.02% NaN_3_ and 3% BSA and incubated for 1 h at room temperature in the dark. Tissues were subsequently washed 3 times with PBS. Coverslips with Prolong Diamond anti-fade mounting media (Thermo) were used for mounting. Immunofluorescent staining was visualized using an inverted fluorescence microscope (Axio Observer A1, Zeiss). The sagittal panorama of the spinal cord was created and exported with the Autopano Giga v3.0.0 software program (Kolor, Lyon, France).

### Quantitative and statistical analysis

For quantitative analysis of transplanted HUMSCs in the spinal cord, HLA^+^ cells (HLA is expressed in only human cells) with a H33258^+^ nucleus were counted throughout the width of each excised spinal cord segment from C7, T9 and L1.

All data are presented as the mean ± SEM. Statistical analysis was performed with Student’s t-test or ANOVA followed by a Bonferroni-Dunn multiple comparisons test (GraphPad Prism 6 software, CA, USA). Differences were considered statistically significant when *P < 0.05.

## Results

### *In vitro* chemotactic migration of HUMSCs towards CGRP

**Figure 1:**
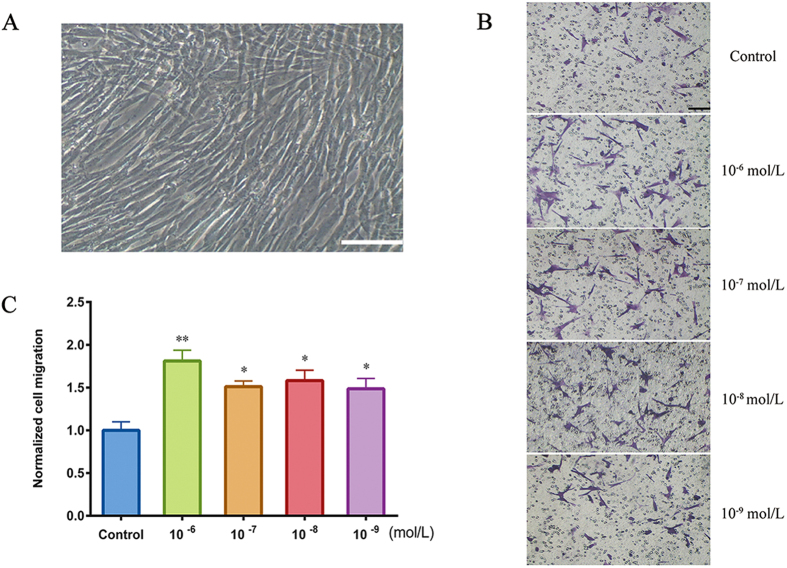
HUMSC morphology and trans-filter chemotactic migration. (**A**) HUMSCs, long spindle-shaped phenotype. Scale bar = 100 μm. (**B**) Trans-filter HUMSCs were stained with cresyl violet after a 6-h induction of 0, 10^−6^, 10^−7^, 10^−8^ and 10^−9^ mol/L CGRP. B: Images are representative of migratory fields on the underside of the membrane. Control cells were incubated in serum-free medium without CGRP. Scale bar = 100 μm. (**C**) CGRP-induced chemotactic migration of HUMSCs compared with control. Cells that migrated to the lower side of the membrane were counted under a phase-contrast light microscope (100×). Values were normalized to the control. Data represent the mean ± SEM from at least 3 independent experiments. *P < 0.05 and **P < 0.01 compared with control.

**Figure 2:**
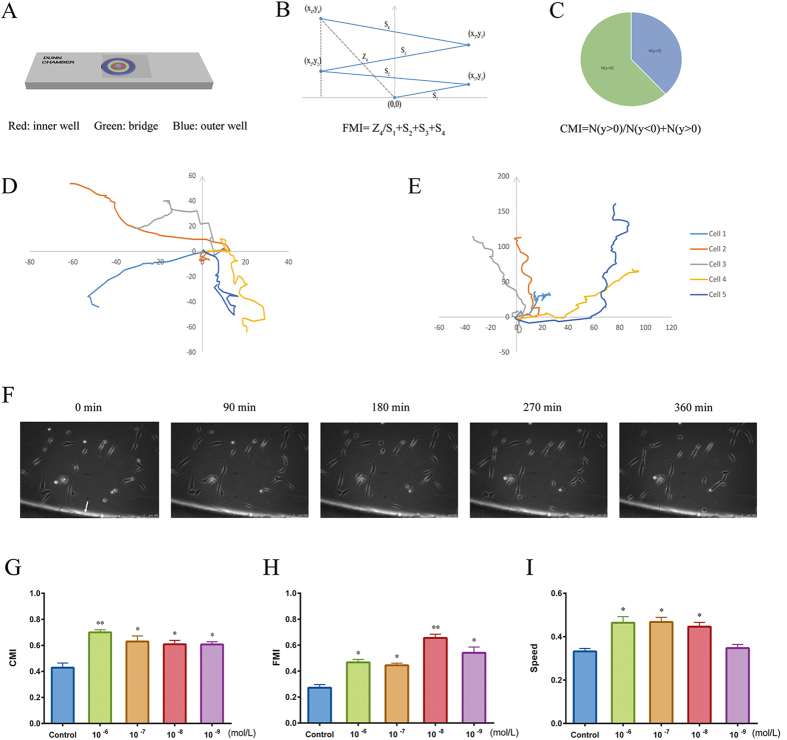
Analysis of CGRP-induced HUMSC chemotaxis with a Dunn chamber assay. (**A**) Schematic model of the Dunn chamber device with the overlying coverslip, showing the position of the inner well (red), bridge (green), and outer well (blue). (**B,C**) Illustrations of FMI (**B**) and CMI (**C**), respectively. (**D,E**) Migration tracks of five representative cells in the absence of CGRP (**D**) and in the presence of 10^−6^ mol/L CGRP (**E**). The starting point for each cell is the intersection between the X- and Y-axes (0, 0), and the arrow represents the direction of the outer well. (**F**) Cells induced by 10^−6^ mol/L CGRP on the bridge between the inner and outer well of the chamber were observed under phase-contrast optics. The arrow points to the outer well of Dunn chamber. HUMSC migration was recorded continuously by time-lapse imaging. (**G–I**) CMI, FMI and migration speed (μm/min) for HUMSCs exposed to varying CGRP concentrations compared with control. Data are shown as the mean ± SEM from at least 3 independent experiments (*P < 0.05 and **P < 0.01).

*In vitro* HUMSC-directed migration was evaluated by a microchemotaxis Boyden chamber. CGRP, at concentrations ranging from 10^−9^ to 10^−6^ mol/L, was added to the lower chamber, and cells that migrated trans-filter to the bottom surface of the membrane were counted. As shown in Fig. 1B,C, HUMSCs exhibited chemotactic movement in the presence of all CGRP concentrations. Compared with other concentrations, 10^−6^ mol/L CGRP produced maximum migration, a nearly 2-fold increase in the cells that crossed the membrane. However, this apparatus can assess only the chemotactic migration of cells as a group. To visualize the migratory behavior of a single HUMSC responding to CGRP and to determine the migratory responses, including the movement tracks, direction (CMI), efficiency (FMI) and migration speed, we used a Dunn chemotaxis chamber^33^. In this device, chemokines are added to the outer well and diffuse across the middle bridge to the inner well, forming a stable linear gradient 30 min after the apparatus is prepared (Fig. 2A–C). In our experiments, we calculated CMI and found increased CMI at each CGRP concentration, and 10^−6^ mol/L CGRP produced the largest increase (Fig. 2G). This result was consistent with that of the Boyden Chamber assay above. Although HUMSCs were attracted to this peptide, a difference in the chemotactic efficiency between each concentration was also observed. As shown in Fig. 2H, 10^−8^ mol/L CGRP possessed a higher FMI than the other concentrations and the control. The migration speed of HUMSCs in the 10^−6^, 10^−7^ and 10^−8^ mol/L groups increased (Fig. 2I), but this increase was not observed in the 10^−9^ mol/L group. In general, 10^−6^ mol/L was an efficient concentration of CGRP for studying *ex vivo* HUMSC migration.

### CGRP 8–37 can block the CGRP-induced chemotactic migration of HUMSCs

**Figure 3:**
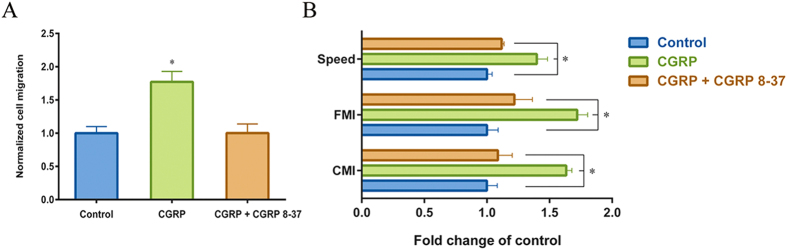
Effects of CGRP 8–37 on CGRP-induced migration of HUMSCs. Cells were pretreated with 10^−6^ mol/L CGRP 8–37 for 30 min, and migration was assessed with the antagonist of CGRP. (**A**) Trans-filter chemotactic migration of HUMSCs assessed using a Boyden chamber. After incubation for 6 h, cells attached to the lower surface of the filter were counted. (**B**) CMI, FMI and migration speed assessed with the Dunn chamber. Values were normalized to the control values (without CGRP stimulation). *P < 0.05 compared with control.

CGRP 8–37 is a specific antagonist of the CGRP receptor. To determine whether CGRP 8–37 can block the CGRP-induced chemotactic migration of HUMSCs, we used *in vitro* Boyden chamber and Dunn chamber assays. Cells were pretreated in 10^−6^ mol/L CGRP 8–37 for 30 min and incubated in serum-free medium containing the same concentration of the antagonist. Approximately 10^−6^ mol/L CGRP was added to the lower compartment of Boyden chamber or the outer well of Dunn chamber. Over 6 h and in the presence of CGRP 8–37, the numbers of HUMSCs that migrated towards CGRP decreased to the level of the control (Fig. 3A), and the elevated levels of CMI, FMI and migration speed induced by CGRP were also abolished (Fig. 3B). These results provided further proof of CGRP induced-chemotaxis in HUMSCs and indicated that CGRP 8–37, with specific blocking effects, could be used for additional *in vivo* experiments.

### CGRP or CGRP 8–37 has no effects on HUMSCs apoptosis, death and proliferation *in vitro*

**Figure 4:**
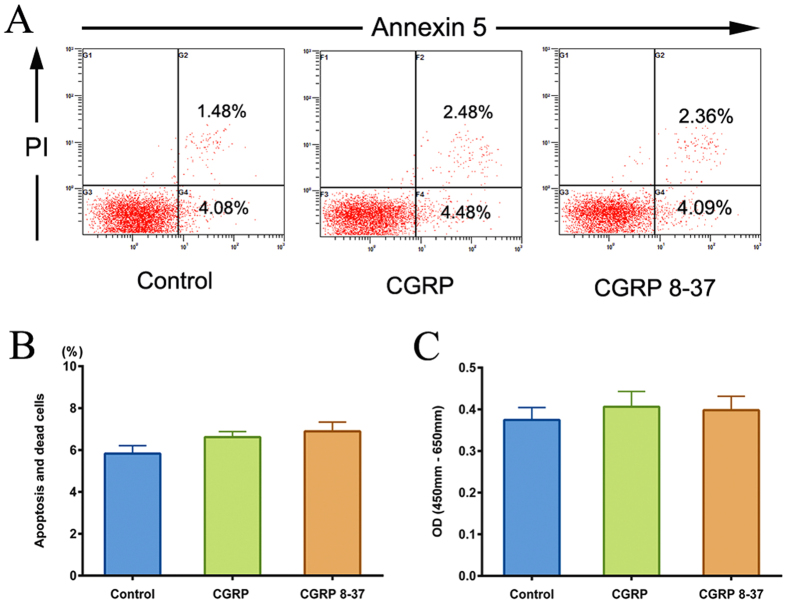
Influence of CGRP or CGRP 8–37 on HUMSCs apoptosis, death and proliferation. (**A**) Apoptotic and dead cells were quantified through FACS analysis after staining with Annexin-V and PI. The Annexin-V+/PI− cells appeared early in the apoptotic process. Cells in death or late apoptosis were Annexin- V+/PI+. (**B**) The percentage of apoptotic and dead cells in CGRP or CGRP 8–37 groups compared with control. Cells in control group were cultured in only L-DMEM containing 10% FBS. (**C**) The CCK-8 proliferation assay showed the OD value after treatment with CGRP or CGRP 8–37 for 7 days. OD value = OD (450 nm)–OD (650 nm). Data are shown as mean ± SEM from at least three independent experiments.

After incubation in medium containing 10^−6^ mol/L CGRP or CGRP 8–37, HUMSCs apoptosis and death rates were measured by detection of Annexin-V/PI staining. The quantitative analysis showed that the percentage of apoptotic and dead cells in CGRP or CGRP 8–37 groups (6.62% and 6.89% respectively) exerted no difference compared with control group (5.83%) (Fig. 4A,B). In addition, CCK-8 assay revealed that treatment with CGRP or CGRP 8–37 for 7 days also had no significant influence on HUMSCs proliferation *in vitro* (Fig. 4C).

### Increase of CGRP in spinal cord tissue following SCI

**Figure 5:**
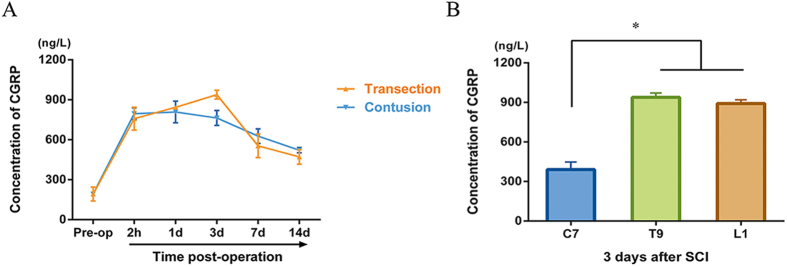
Changes in CGRP concentrations in the parenchyma after SCI. (**A**) CGRP concentrations over time in the T9 segments of different injury models. Pre-op represents the average CGRP levels at the same segment in rats without any surgery. (**B**) Depositions of CGRP throughout the spinal cord after SCI. At 3 d post-transection surgery, the C7, T9 and L1 segments were excised for analysis. Data are presented as the mean ± SEM. *P < 0.05 versus C7.

CGRP concentrations in spinal cord tissue were detected by ELISA. As demonstrated in Fig. 5A, the CGRP concentration in the T9 segment of preoperative rats remained at 192.3 ng/L, with a sharp increase to more than 750 ng/L at 2 h post-operation both in the transection and contusion SCI rats. CGRP concentrations decreased with time in non-transected rats, while the concentration peaked at 938.5 ng/L 3 d post-transection. During the entire observation period after SCI, CGRP concentrations in the T9 segment were elevated compared with baseline levels. We then tested the CGRP concentrations in spinal cord parenchyma at different sites 3 d post-transection and found that the CGRP concentrations at the T9 (injury site) and L1 (caudal) segments were elevated compared with that in the C7 (rostral) segment (Fig. 5B). However, no significant variance was observed between the concentrations detected in the T9 and L1 segments.

### CGRP 8–37 decreased HUMSC homing to the injury site

**Figure 6:**
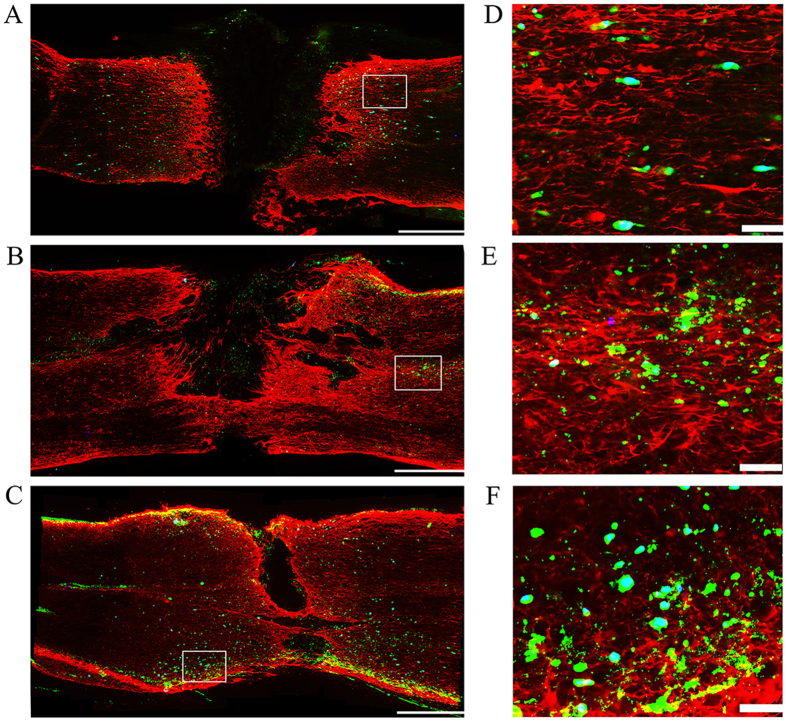
Immunohistochemistry showing transected SCI lesion sites in different groups. (**A**) Control group; (**B**) CGRP 8–37 group; (**C**) CGRP group. Sections were double-stained for GFAP (red) and HLA (green) 7 d after transplantation. The GFAP-devoid area represents the glial scar after SCI. Engrafted HUMSCs (green with a blue nucleus) were located around the border of the scar. Left to right: rostral-caudal direction. Scale bar = 500 μm. (**D–F**) Magnification of the area marked with a rectangle in (**A–C**) respectively. Scale bar = 50 μm.

**Figure 7:**
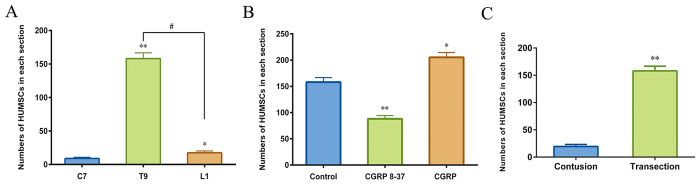
Distribution of transplanted HUMSCs after SCI. (**A**) Cell distribution along the spinal cord after transection in the control group. Three regions were examined, C7 (rostral), T9 (injury site) and L1 (caudal). (**B**) Cells in the T9 (transection lesion site) segment of different groups (with continuous PBS, CGRP 8–37 and CGRP at the injury sites). (**C**) HUMSCs located at the lesion sites of different SCI models. HLA^+^ and H33258^+^ double-stained cells in the parenchyma of the spinal cord were counted for each tissue section. *P < 0.05 and **P < 0.01 compared with the control (**A**), C7 (**B**) and contusion (**C**) groups. ^#^P < 0.01.

To better analyze the long-distance migration of cells and to prevent cell loss after injection, LP was selected in lieu of intravascular or direct parenchymal injections. HUMSCs labeled with Hoechst 33258 *in vitro* were transplanted, and HLA immunohistochemical staining further identified the grafted human cells. A tube was placed at the T9 segment to continuously administer PBS, CGRP 8–37 or CGRP. After a long homing journey, the majority of HUMSCs were detected at the borders of GFAP-devoid scars (Fig. 6A–C), whereas the only cells in the C7 and L1 segments tended to surround the dura (data not shown). As shown in Fig. 7A, transplanted HUMSCs migrated preferentially to the injury site (158.1 ± 8.6 cells per section) and rarely reached the rostral and caudal ends (9.0 ± 1.8 cells and 17.4 ± 2.8 cells, respectively). With CGRP 8–37 treatment, nearly half of HUMSCs failed to reach the injury site (88.07 ± 6.1 cells). In the CGRP group, we noticed an increase of HUMSCs at the epicenter compared with controls (Fig. 7B). Meanwhile, we investigated how HUMSCs migrated to the lesion site of different types of SCI and found that very few HUMSCs were observed in the parenchyma of the injury site (19.38 ± 3.7 cells) in the contusion group compared with those of the transection group (158.1 ± 8.6 cells) (Fig. 7C).

### PI3K/Akt and MAPK signaling pathways play a critical role in CGRP-induced HUMSC migration

**Figure 8:**
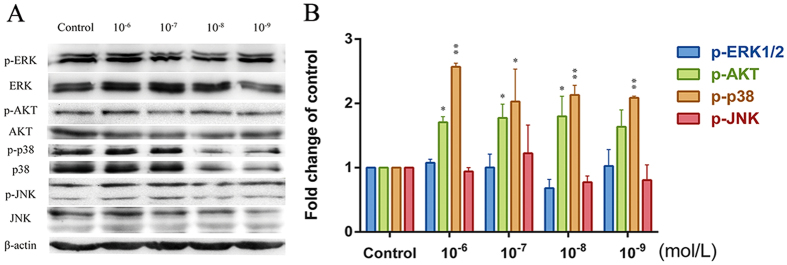
MAPK and PI3K/Akt phosphorylation in HUMSCs after CGRP stimulation. HUMSCs were stimulated with various concentrations (0, 10^−6^, 10^−7^, 10^−8^ and 10^−9^ mol/L) of CGRP for 6 h. The active phosphorylated forms of ERK1/2, Akt, p38MAPK and SAPK/JNK (p-ERK1/2, p-Akt, p-p38MAPK, and p-SAPK/JNK) and the total proteins (ERK1/2, Akt, p38MAPK, and SAPK/JNK) were measured by densitometry using the Image Lab 4.1 software. The density ratio of phosphorylated to total ERK1/2, Akt, p38MAPK and SAPK/JNK was normalized to its related control. Equal protein loading was assessed with β-actin levels. Data are presented as the mean ± SEM. *P < 0.05 and **P < 0.01 compared with the respective CGRP-unstimulated HUMSCs.

**Figure 9:**
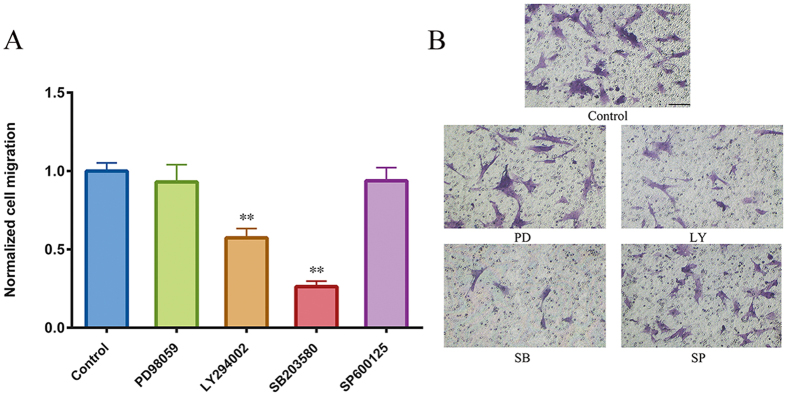
Effects of PD98059, LY294002, SB203580, or SP600125 on the CGRP-induced chemotaxis of HUMSCs. After pretreatment for 30 min, HUMSCs were added to the upper compartments of the Boyden chamber in the presence of the indicated inhibitors. (**A**) Cells that had migrated to the lower side of the filter, representative images in (**B**), were counted 6 h later. Control represents the cells only attracted to CGRP. **P < 0.01 compared with control. Scale bar = 100 μm.

We next examined MAPK (ERK1/2, SAPK/JNK, and p38MAPK) and Akt phosphorylation in HUMSCs at different CGRP concentrations (Fig. 8A,B). Treatment with CGRP (0, 10^−6^, 10^−7^, 10^−8^ and 10^−9^ mol/L) for 6 h did not alter ERK1/2 and SAPK/JNK phosphorylation but led to concentration-dependent p38MAPK phosphorylation (peaking at 10^−6^ mol/L CGRP). Akt phosphorylation was elevated following stimulation with 10^−6^, 10^−7^, 10^−8^ mol/L CGRP but not in the 10^−9^ group. Thus, subsequent experiments were performed at 10^−6^ mol/L to examine which pathways were involved in CGRP induced-chemotaxis. HUMSCs were pretreated for 30 min with 3 × 10^−5^ mol/L LY294002 (PI3K/Akt inhibitor), 3 × 10^−5^ mol/L SB203580 (p38MAPK inhibitor), 5 × 10^−5^ mol/L PD98059 (ERK1/2 inhibitor), or 10^−5^ mol/L SP600125 (SAPK/JNK inhibitor), and their migratory responses to 10^−6^ mol/L CGRP in the presence or absence of inhibitors were examined by a microchemotaxis Boyden chamber migration assay. As shown in Fig. 9A,B, the CGRP-induced chemotactic migration of HUMSCs suffered a sharp decline when the PI3K/Akt and p38MAPK pathways were inhibited by LY294002 and SB203580, respectively. A more obvious inhibition was produced by SB203580. Abolishment of the ERK1/2 and SAPK/JNK signaling pathways with PD98059 and SP600125, respectively, had no significant influence on CGRP-induced migration.

### The role of Akt or p38MAPK signaling pathway in HUMSCs apoptosis, death and proliferation with the presence of CGRP

**Figure 10:**
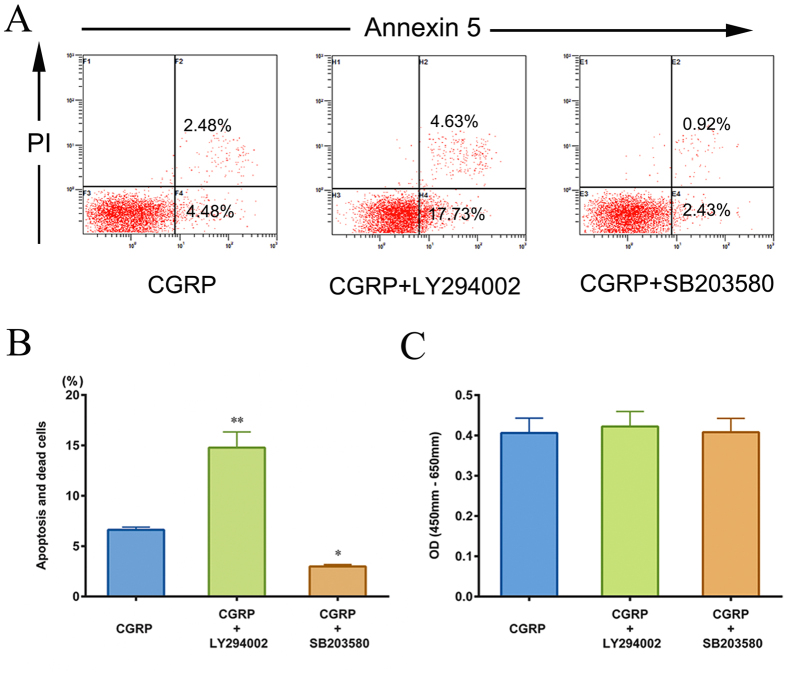
Influence of LY294002 or SB203580 on HUMSCs apoptosis, death and proliferation with the presence of CGRP. (**A**) Apoptosis and death of HUMSCs were assessed through FACS analysis. (**B**) By inhibition of Akt or p38MAPK, the percentage of apoptosis and dead cells changed. (**C**) The proliferative capacity of HUMSCs in each group was calculated using a CCK-8 assay, and the results were differently displayed on OD value columns. Data are shown as mean ± SEM from at least three independent experiments. **P < 0.01, *P < 0.05 compared with CGRP group.

To speculate the mechanism of activated Akt and p38MAPK in regard to HUMSC survival, Annexin-V/PI and CCK-8 assay were taken to verify the influence of LY294002 or SB203580 on HUMSCs apoptosis, death and proliferation with the presence of CGRP. Figure 10A,B revealed that the number of apoptosis and dead cells increased dramatically by inhibition of Akt (14.78% vs. 6.62%). However, the inhibitor of p38 MAPK, SB203580, aided CGRP protecting HUMSCs from apoptosis and death (2.99% vs. 6.62%). The CCK-8 proliferation assay indicated that blockage of Akt or p38MAPK pathway did not change the effect of CGRP on HUMSCs proliferation (Fig. 10C).

## Discussion

HUMSCs, as a hopeful candidate for treating SCI^34^, possess many advantages. Transplanted MSCs via LP, intravascular or direct parenchymal injections all exhibit preferential homing to the injury site, and chemokines released from the lesion site contribute to this process^13^,^14^,^35^. However, the critical factors that promote MSC homing after SCI are still not well understood. CGRP is synthesized in the spinal cord and mediates sensory transmission, inflammation, immunomodulation and tissue healing after tissue injury^22^,^36^,^37^. Thus, we supposed that secreted CGRP after SCI may participate in therapeutic MSC homing. To avoid disturbances of the complex environment *in vivo*, we first evaluated the CGRP-induced chemotactic migration of HUMSCs *in vitro* using the Boyden chamber and Dunn chamber. HUMSCs exhibited chemotaxis in the presence of CGRP, which increased the CMI and FMI of HUMSCs and accelerated their migration speed. The abolishment of CGRP-induced migration with CGRP 8–37, a specific antagonist of CGRP, further demonstrated the function of CGRP on HUMSC migration.

Consistent with previous studies on the changes in CGRP after SCI^25^, our data showed an increase in CGRP at the injury site for 2 weeks after the 2 types of injury. The CGRP concentrations in the T9 or L1 segments were much higher than that in the C7 segment at 3 d post-transection. This observation is a result of biochemically induced events in response to spinal lesions, where more CGRP-containing primary afferents sprout intraspinally, and upregulated CGRP from DRG neurons is transferred into inflammatory sites. The ascending deafferentation followed by T9 damage contributed to the aggradation of CGRP at the lesion and caudal sites. As CGRP peaked at 3 d post-transection, this time point was used for cell transplantation experiments.

Next, with the artificial manipulation of the microenvironments at the lesion sites, we confirmed that CGRP acted as a key chemokine to mediate HUMSCs homing after SCI. Stem cells were preferentially localized to the injured region under the stimulation of this factor, which could be reduced with a competitive inhibitor of this peptide. In SCI rats at 7 d post-transplantation, the majority of HUMSCs were at the GFAP border, avoiding glial scars, and only a small number of transplanted cells remained in cervical and caudal regions. In contrast, a previous report on the distribution of LP-transplanted MSCs indicated that more cells were located in the L3-L5 (injection site) segments compared with those at the T8 (injury site) segment. These contradictions could be explained by the differences in the type of SCI and variations in the tissues analyzed. We assessed the distribution of HUMSCs in the spinal parenchyma of a transected SCI model, whereas researchers in that group discussed the location of MSCs not only in the parenchyma but also in the intrathecal space of clip impact-compression SCI rats. Meanwhile, we found a discrepancy between the distributions of CGRP and HUMSCs along the spinal cord. Increase of CGRP did not recruit HUMSCs in L1 segment. And relative homing to the injured site was not seen in contusion SCI rats. Together, we consider that the disruption of the dura was required for the release of CGRP to promote the homing of engrafted cells. Though there is no difference of increased CGRP in injury sites of these two SCI models, transection model which represents a more severe injury lead to a larger release of other chemokines^38^ that may help CGRP recruit more HUMSCs sequentially. The disrupted dura in the transection model allows more accumulation and penetration of CGRP to the surrounding pia of the injured spinal cord, which is very likely to cause a physical blockage interrupting the passage of the intrathecal cells. In conclusion, we demonstrated that HUMSCs migrated preferentially to sites of injury in the transection group and that cell transplantation via LP is more plausible for use in section SCI.

The binding of CGRP to its cognate receptor on MSCs^29^ was thought to facilitate conformational changes in the receptor that led to the activation of downstream signaling pathways^23^. Because of the increasing clinical significance of CGRP, the signal transduction pathways involved in the chemotactic migration of HUMSCs should be studied. The atypical CGRP receptor is composed of a distinct assembly, a G protein-coupled receptor, a single transmembrane protein, and an additional protein required for intracellular Gα_s_ and β-arrestin coupling^39^,^40^,^41^,^42^,^43^,^44^. β-arrestin can associate with CGRP receptors and may aid the activation of PI3K/Akt and MAPK (ERK1/2, p38MAPK and SAPK/JNK) cascades involved in many critical physiological processes^45^,^46^,^47^. In the present study, we found that Akt and p38MAPK phosphorylation in HUMSCs was increased to varying degrees by CGRP stimulation, which was not observed in the ERK1/2 and SAPK/JNK pathways. By inhibiting MAPK or Akt pathways in the Boyden chamber assay, we sought to clarify whether these signaling molecules were involved in the CGRP-induced chemotaxis of HUMSCs. Treatment with LY294002 and SB203580 obviously reduced the number of migrating cells, indicating that Akt and p38MAPK played a role in CGRP-induced migration. These findings deepen our understanding of how CGRP attracts HUMSCs and may facilitate the improvement of the migratory efficiency of MSCs to CGRP via their modification.

To exclude the possibility that increase in HUMSCs at the epicenter induced by CGRP may be a reflection of the increased viability of HUMSCs, not entirely from migration. We did test *in vitro* as it is full of distractions to clarify the function of CGRP on cell survival *in vivo*, CCK-8 and Annexin V-FITC apoptosis assay revealed that exogenous CGRP or CGRP 8–37 has no effects on HUMSCs proliferation, apoptosis and death. Moreover, no obvious HUMSCs that underwent apoptosis *in vivo* were observed according to the morphology of nucleus stained by Hoechst in Fig. 6. The results together could eliminate the influence of CGRP on cell survival, which delineate that the increased HUMSC at the epicenter under the influence of CGRP is directly related to chemotactic migration. A previous study reported that CGRP overexpression would reduce death and apoptosis of rat adipose-derived stem cells and promote cell proliferation^31^. We attribute the divergences to whether CGRP is endogenous or exogenous and the different types of cells. Endogenous CGRP may activate different signaling pathways leading to that results.

Since Akt and p38MAPK are known as the canonical signaling pathways that participate in the regulation of cell apoptosis^48^,^49^, we also explored why activation of Akt and p38MAPK by CGRP fails to mediate a change on HUMSCs proliferation, apoptosis and death. Our results showed that in the presence of LY294002, the number of apoptotic and dead cells increased dramatically. However, the inhibitor of p38MAPK, SB203580, helped CGRP protect HUMSCs from apoptosis and death. As presented in Fig. 4, CGRP did not influence HUMSCs apoptosis and death, it is inferred that the antagonistic effects of these two pathways give rise to CGRP’s inefficiency on HUMSCs survival. Our previous study has confirmed that activated PI3K/Akt and p38MAPK signaling pathways are involved in chemotactic migration of MSCs^32^. The co-activation of Akt and p38 MAPK could synergistically regulate dynamics of FAs and rearrangement of cytoskeleton promoting HUMSCs chemotactic migration.

Currently, accumulating evidence recommends the possible use of MSCs in the treatment of brain or spinal cord disorders^50^,^51^. These cells respond to secreted factors and migrate toward the site of injury. However, only a small subpopulation successfully reaches the damaged areas^52^,^53^, limiting the curative effect of MSCs. Here, we found that CGRP is a key chemokine facilitating HUMSC homing after SCI and that this peptide can easily induce the *in vitro* chemotactic migration of HUMSCs by activating the PI3K/Akt and MAPK signaling pathways. Thus, future efforts should use CGRP as a model chemokine *ex vivo* to enhance the migratory efficiency of transplanted MSCs after SCI. More intensive studies are required to further delineate whether modified CGRP-sensitive HUMSCs are directed to the lesion site *in vivo* and to determine the contribution of HUMSCs to neuronal regeneration and functional restoration in injured rats. Because higher CGRP expression is observed at the site of SCI, its other properties should be further verified. Our preliminary results reveal that the expression of Map2, MBP and Nestin in CGRP-induced HUMSCs is significantly higher than that in the control groups. The morphology of cells in CGRP groups also develops into characteristic round cell bodies, with more branching extensions, bipolar or multipolar in shape (S. Fig. 1). CGRP could promote neural differentiation of HUMSCs. As cell delivery via LP is more efficient in transected SCI rats, whether this strategy can be used therapeutically for patients needs to be evaluated.

## Additional Information

**How to cite this article**: Zhang, Y. *et al.* Calcitonin gene-related peptide is a key factor in the homing of transplanted human MSCs to sites of spinal cord injury. *Sci. Rep.* **6**, 27724; doi: 10.1038/srep27724 (2016).

## Supplementary Material

Supplementary Information
