# Supplementary material for: Calcitonin gene-related peptide is a key factor in the homing of transplanted human MSCs to sites of spinal cord injury
Source: Sci Rep. 2016 Jun 14;6:27724. doi: 10.1038/srep27724 (PMC4906351; doi:10.1038/srep27724)
Supplement: Supplementary Information [file srep27724-s1.pdf]

Supplementary Information

Calcitonin gene-related peptide is a key factor in the homing of transplanted human MSCs to sites of spinal cord injury

Yu Zhang[1,*], Jinhua Yang[2,*], Peng Zhang[1], Tao Liu[1], Jianwei Xu[3], Zhihai Fan[1], Yixin Shen[1], Wenjie Li[4] & Huanxiang Zhang[3]

[1]Department of Orthopedics, the Second Affiliated Hospital of Soochow University, Suzhou, China.

[2]Department of Orthopedics, the Second People's Hospital of Changshu, Suzhou, China.

[3]Department of Cell Biology, Jiangsu Key Laboratory of Stem Cell Research, Medical College of Soochow University, Suzhou Industrial Park, Suzhou, China.

[4]Department of Human Anatomy, Medical College of Soochow University, Suzhou Industrial Park, Suzhou, China.

*These authors contributed equally to this work.

Supplementary Materials and Methods

In vitro differentiation of HUMSCs to neural cells

To identify the functional role of CGRP on HUMSCs differentiation, HUMSCs were seeded at a density of 2×10^4 cells/cm^2 on 0.01% poly-L-lysine (PLL; Sigma)-coated dishes. When cells reached approximately 50% confluence, neural differentiation was performed. Cells in CGRP group were grown in Neurobasal medium (containing 1% N_2, Gibco) supplemented with 10^{-7} mol/L CGRP (Sigma). And cells in Control group were cultured only in Neurobasal medium with same volume PBS. Approximately 75% of the media was replaced every 3 days. These cells were examined 10 days after differentiation using immunocytochemistry for specific neuro-markers. Map2: a protein found specifically in dendritic branching of neuron; MBP: a protein recognized as the marker of oligodendrocyte or Schwann's cell; Nestin: a protein specifically expressed in neuroepithelial stem cells[1].

Immunocytochemistry

HUMSCs were fixed in cold 4% paraformaldehyde in 0.1M PBS (pH 7.2) overnight, then washed three times with PBS (5 min) and incubated with primary Abs overnight at 4℃. Primary Abs were diluted in PBS/0.02% NaN_3/3% bovine serum albumin (BSA)/0.2% Triton X-100 at the following working concentrations: mouse mAb anti-nestin, 1:200 dilution (Chemicon, Temecula, CA); mouse mAb anti-Map2, 1:200 dilution

(Sigma); rabbit mAb anti-MBP, 1:200 dilution (Abcam). After incubation of primary Abs, cells were rinsed with PBS three times prior to secondary antibody application. FITC-conjugated goat anti-mouse Abs (Invitrogen), Cy3-conjugated goat anti-rabbit Abs (Invitrogen) were diluted 1:150 in PBS/0.02% NaN_3/3% BSA and applied to cells for 1 h at room temperature in the dark. Cells were subsequently washed in PBS three times, and cell nuclei were counterstained with Hoechst 33258 (1:100; Sigma). Fluorescence was examined with Leica DMI 6000 B microscope (Germany). Cells treated with nonspecific mouse IgM, or secondary Abs alone showed no staining.

Supplementary Figure

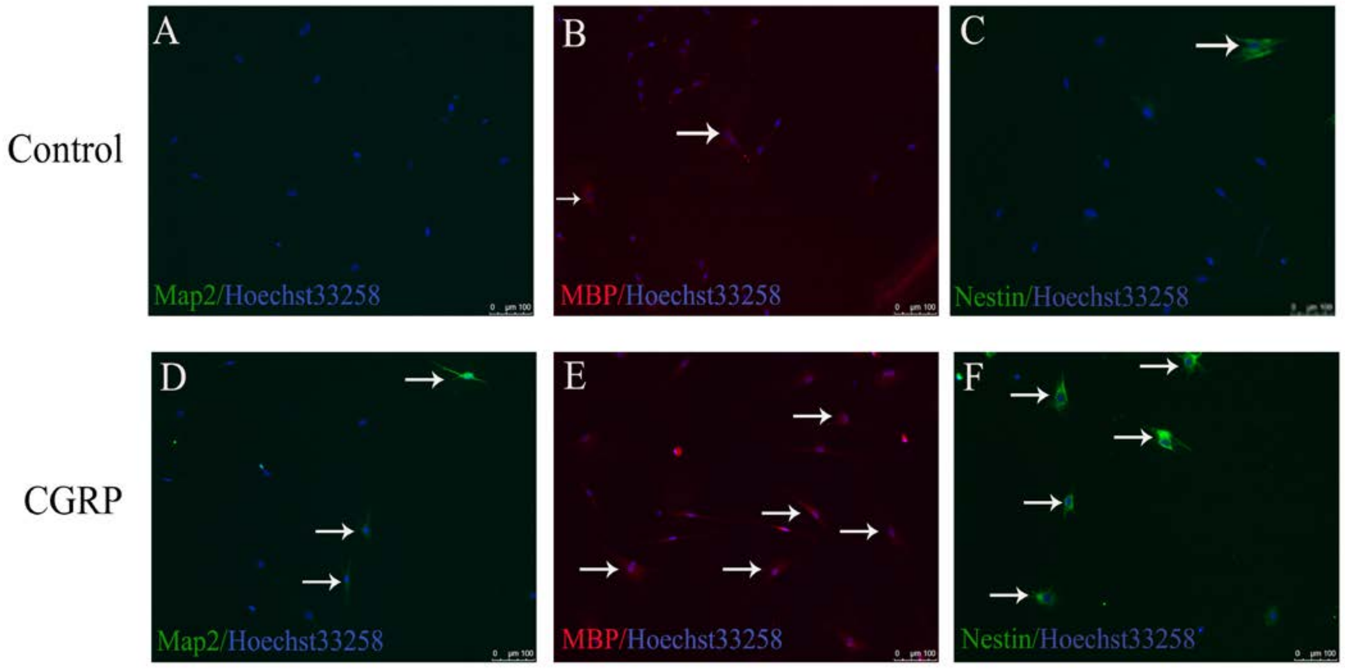

Supplementary Figure S1. Neural markers expression by immunostaining. Immuostaining analysis to detect the expressions of various neural protein markers after CGRP induction for 10 days (D, E and F). Cells in control group were cultured in Neurobasal medium added with same volume PBS (A, B and C). A, D: expression of Map2 (green); B, E: expression of MBP (red); C, F: expression of Nestin (green). Nucleus were stained by Hoechst 33258 (blue). Arrows indicate the typical positive cells. Scale bars = 100 µm.

Supplementary References

1 Messerli, M. et al. Stem cells from umbilical cord Wharton's jelly from preterm birth have neuroglial differentiation potential. *Reprod Sci* **20**, 1455-1464 (2013).
